# Rivaroxaban and Hemostasis in Emergency Care

**DOI:** 10.1155/2014/935474

**Published:** 2014-02-20

**Authors:** Jürgen Koscielny, Edita Rutkauskaite

**Affiliations:** Institute for Transfusion Medicine, Charité—Universitätsmedizin Berlin, Charitéplatz 1, 10117 Berlin, Germany

## Abstract

Rivaroxaban is an oral, direct Factor Xa inhibitor, approved for the prevention and treatment of several thromboembolic disorders. Rivaroxaban does not require routine coagulation monitoring and has a short half-life. However, confirmation of rivaroxaban levels may be required in circumstances such as life-threatening bleeding or perioperative management. Here, we explore the management strategies in patients receiving rivaroxaban who have a bleeding emergency or require emergency surgery. Rivaroxaban plasma concentrations can be assessed quantitatively using anti-Factor Xa chromogenic assays, or qualitatively using prothrombin time assays (using rivaroxaban-sensitive reagents). In patients receiving long-term rivaroxaban therapy who require elective surgery, discontinuation of rivaroxaban 20–30 hours beforehand is normally sufficient to minimize bleeding risk. For emergency surgery, we advise against prophylactic use of hemostatic blood products, even with high rivaroxaban concentrations. Temporary rivaroxaban discontinuation is recommended if minor bleeding occurs; for severe bleeding, rivaroxaban withdrawal may be necessary, along with compression or appropriate surgical treatment. Supportive measures such as blood product administration might be beneficial. Life-threatening bleeding demands comprehensive hemostasis management, including potential use of agents such as prothrombin complex concentrate. Patients taking rivaroxaban who require emergency care for bleeding or surgery can be managed using established protocols and individualized assessment.

## 1. Introduction

Rivaroxaban is an oral, direct Factor Xa inhibitor that has been developed in recent years. It is a selective inhibitor of free Factor Xa, as well as Factor Xa bound in the prothrombinase complex or associated with thrombin [1]. Rivaroxaban has a high oral bioavailability, a rapid onset of action, and few drug–drug interactions, and it requires no dose adjustment in terms of age, sex, or body weight [1, 2]. The half-life of rivaroxaban is 5–13 hours (5–9 hours in healthy individuals; 11–13 hours in the elderly) [2–4]. After administration, two-thirds of the rivaroxaban dose is metabolized in the liver (via cytochrome P450 [CYP] 3A4, CYP2J2, and CYP-independent biotransformation); approximately half of this inactive product is then excreted through the kidneys and the remainder in the feces. The remaining one-third of the dose is eliminated as unchanged drug by the kidneys [2]. In addition, rivaroxaban has no major or active circulating metabolites [2, 5, 6]. Rivaroxaban is not recommended in patients with severe renal failure (creatinine clearance [CrCl] < 15 mL/min) or in patients with hepatic disease associated with coagulopathy and clinically relevant bleeding risk, including cirrhotic patients classified as Child-Pugh B or C [2].

Rivaroxaban is approved in many countries worldwide for the prevention of venous thromboembolism in patients undergoing elective hip or knee replacement surgery, for stroke prevention in patients with nonvalvular atrial fibrillation, and for the treatment and secondary prevention of recurrent deep vein thrombosis and pulmonary embolism [2, 7]. Rivaroxaban has also been granted approval in Europe for secondary prevention of atherothrombotic events in adult patients who have had biomarker-confirmed acute coronary syndrome, in combination with standard antiplatelet therapy [2].

Rivaroxaban has predictable pharmacokinetics and pharmacodynamics and does not require dose adjustment or routine coagulation monitoring [1, 4, 8]. All phase III studies were conducted without routine laboratory testing of the anticoagulant effects of rivaroxaban [9–13], further supporting this approach. Nevertheless, practicing physicians require clinical recommendations for handling emergencies, such as life-threatening bleeding events or emergency surgery, in patients receiving long-term rivaroxaban therapy [14]. In these situations, practical questions arise, including when and which laboratory test(s) should be performed (and whether tests should be qualitative or quantitative)?, when and for how long should rivaroxaban be discontinued?, and how can rivaroxaban-related bleeding be managed?

There are currently no specific reversal agents for either direct thrombin inhibitors (such as dabigatran) or direct Factor Xa inhibitors (such as rivaroxaban and apixaban). In addition, there are no prospective, randomized clinical trials or registry data for patients who experience acute bleeding while receiving these agents, and there is a subsequent lack of evidence-based recommendations or guidelines for physicians. There has also been a lack of randomized clinical trials and real-world studies assessing these bleeding situations in patients receiving traditional anticoagulants, such as vitamin K antagonists (VKAs) or heparins. Even though data have shown that four-factor prothrombin complex concentrates (PCCs) are effective and well tolerated in the reversal of VKA activity in a phase III randomized trial and in daily clinical practice [15, 16], it has been suggested that PCC administration is still suboptimal in emergency situations for VKA reversal, with suitable treatment being administered in only 26% of cases [17].

The current approach for anticoagulant reversal is based primarily on recent experience or on preliminary data from the literature. In terms of patient safety during possible reversal of anticoagulation using drugs or blood products, any increase in thromboembolic risk must be considered. In this paper, we aim to provide guidance on the management of patients who are receiving anticoagulation with rivaroxaban and who may require an emergency intervention. We discuss several topics, including the measurement of rivaroxaban concentrations, approaches in patients with severe or life-threatening bleeding, reversal of the anticoagulant effect, and how bleeding risk may be calculated.

## 2. Laboratory Testing of Rivaroxaban

### 2.1. Qualitative Assessment of Rivaroxaban Using Prothrombin Time

Rivaroxaban affects global coagulation tests, such as the prothrombin time (PT) and activated partial thromboplastin time (aPTT), to a varying degree. Because of the variable sensitivity of aPTT assays to rivaroxaban [18], aPTT is regarded as unsuitable for determining the pharmacodynamic effect of rivaroxaban [2, 14, 19].

The PT is more sensitive than the aPTT [20] and is prolonged in a concentration-dependent manner when sensitive thromboplastin reagents such as STA Neoplastine CI Plus (Diagnostica Stago, Asnières-sur-Seine, France) are used [4, 21, 22]. However, when interpreting prolongation times (seconds), the significant interindividual variability and its relation to the timing of the last dose must be considered [14, 22]. An example of the effect of various rivaroxaban doses on PT using STA Neoplastine CI Plus is shown in Table 1 [2]. However, PT is not useful as a predictor of potential bleeding events. For example, a post hoc analysis of patients undergoing elective hip or knee replacement surgery who received 10 mg rivaroxaban once daily (od) showed no correlation between PT values and bleeding events [23]. Nevertheless, in an acute situation, the determination of PT could deliver valuable preliminary information on the effect of rivaroxaban. A normal PT value, obtained using a sensitive thromboplastin reagent, indicates that a clinically significant residual effect of rivaroxaban is unlikely.

### 2.2. Measurement of Rivaroxaban Plasma Concentration Using an Anti-Factor Xa Test

Anti-Factor Xa assays with chromogenic substrates are routinely available for low molecular weight heparins. With appropriate rivaroxaban calibrators and controls, this type of assay is also suitable for measuring rivaroxaban levels and has been found to be sensitive and specific to rivaroxaban across a wide range of plasma concentrations (20–662 ng/mL) [24].

Assays that do not require the addition of exogenous antithrombin should be used, because false-positive results may otherwise occur, especially in the determination of rivaroxaban trough levels [24].

Rivaroxaban plasma concentrations determined by anti-Factor Xa assays correlate well with those obtained by high-performance liquid chromatography [25]. Using chromogenic anti-Factor Xa assays in patients who had received 20 mg rivaroxaban od for the treatment of acute deep vein thrombosis, a mean peak concentration of 215 ng/mL (range: 22–535 ng/mL) after 2–4 hours and a mean trough level of 32 ng/mL (6–239 ng/mL) after 24 hours were observed [2].

### 2.3. Key Considerations for Laboratory Testing of Rivaroxaban

At least four factors are important in understanding the effects of rivaroxaban on various coagulation parameters. First, knowing when the last dose of rivaroxaban was administered (in relation to the time of blood collection) is critical for interpretation of coagulation data. Owing to the short half-life of rivaroxaban, coagulation tests should be performed and interpreted promptly; otherwise, the data obtained may no longer be clinically applicable. Second, global clotting tests (e.g., PT) are not suitable for quantitative determination of rivaroxaban concentrations. Third, when performing PT testing in an emergency situation, the sensitivity to rivaroxaban of the thromboplastin reagent used must be considered when making a qualitative evaluation of the residual anticoagulant effect. Finally, using anti-Factor Xa assays, quantitative measurement can be made by determining the exact rivaroxaban plasma concentration. A calibration curve generated using rivaroxaban calibrators spanning a range of 0–500 ng/mL should be used, to detect even high levels of rivaroxaban at the maximum effect. The determination of plasma concentrations (e.g., trough levels) could be useful to exclude the possibility of rivaroxaban accumulation in patients, such as those with acute renal or hepatic failure.

## 3. Bleeding Outcomes in Patients Receiving Long-Term Anticoagulation with Rivaroxaban: Experience from ROCKET AF

The phase III ROCKET AF (Rivaroxaban Once Daily Oral Direct Factor Xa Inhibition Compared with Vitamin K Antagonism for Prevention of Stroke and Embolism Trial in Atrial Fibrillation) study compared rivaroxaban (20 mg od; 15 mg od in patients with CrCl 30–49 mL/min) with dose-adjusted warfarin for stroke prevention in patients with nonvalvular atrial fibrillation [12]. The principal safety outcome, defined as a combination of major and nonmajor clinically relevant bleeding events, occurred with a similar incidence in both groups (14.9% per year with rivaroxaban versus 14.5% per year with warfarin); the rates of major bleeding were also similar (3.6% per year versus 3.4% per year). A major bleeding event was defined as clinically overt bleeding in association with a hemoglobin decrease of 2 g/dL or more, transfusion of two or more units of red blood cell concentrates or whole blood, bleeding in a critical location (intracranial, intraspinal, intraocular, pericardial, intra-articular, intramuscular with compartment syndrome, or retroperitoneal), or fatal bleeding. The incidence of gastrointestinal bleeding was significantly higher with rivaroxaban therapy compared with warfarin (3.2% versus 2.2%; *P* < 0.001). However, rivaroxaban was associated with significantly fewer fatal bleeding events compared with warfarin (0.2% per year versus 0.5% per year; *P* = 0.003) and significantly fewer cases of intracranial hemorrhage (0.5% per year versus 0.7% per year; *P* = 0.02) [12].

In ROCKET AF, major bleeding events in patients receiving rivaroxaban occurred predominantly in patients with preexisting conditions of the gastrointestinal tract. Bleeding management was based primarily on the underlying cause of bleeding usually sufficient to manage these events.

## 4. Assessment of Bleeding Risk and Clinical Management of Bleeding Events

### 4.1. Elective Surgery

In patients with normal renal and hepatic function scheduled to undergo elective surgery, discontinuation of rivaroxaban at least 24 hours before the operation is sufficient to normalize a drug-related risk of bleeding [2]. A “rule of thumb” is to allow a period of twice the rivaroxaban half-life to elapse because, after this period, the residual plasma concentration of rivaroxaban is lower and does not exert any relevant pharmacodynamic effects. However, risk of bleeding in relation to timing of catheter removal in patients receiving epidural analgesia, as well as the risk of intraoperative bleeding associated with each procedure, should be taken into account. Bleeding in the mucosa or in larger body cavities is more difficult to evaluate than, for example, bleeding during and after neurosurgical operations.

**Figure 1:**
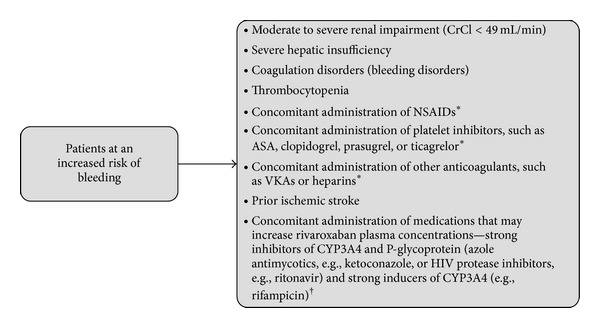
Factors associated with increased risk of bleeding with rivaroxaban [2]. *Care is to be taken in patients receiving concomitant systemic treatment with these agents and rivaroxaban. ^†^Rivaroxaban is not recommended in patients receiving concomitant systemic treatment with these agents. ASA = acetylsalicylic acid; CrCl = creatinine clearance; CYP3A4 = cytochrome P450. 3A4; HIV = human immunodeficiency virus; NSAID = nonsteroidal anti-inflammatory drug; VKA = vitamin K antagonist.

Several factors can lead to an increased level of rivaroxaban in plasma and a greater risk of bleeding. Rivaroxaban use in a phase I study in subjects with moderate renal impairment (CrCl 30–49 mL/min) was associated with an increase in plasma concentration (area under the plasma concentration-time curve [AUC] increased 1.5-fold) [26]. In addition, age >75 years (AUC increased 1.4-fold) and moderate hepatic impairment (AUC increased 2.3-fold) can lead to greater exposure [27, 28]. Unlike with dabigatran and apixaban, no gender effect was observed in relation to the plasma concentration of rivaroxaban [2, 29, 30]. When rivaroxaban is coadministered with other anticoagulants, an increased risk of bleeding must be assumed. Potential predictors for the increased risk of bleeding with rivaroxaban are listed in Figure 1 [2].

### 4.2. Emergency Surgery

With greater use of the direct oral anticoagulants, the number of patients who require emergency surgery while receiving these agents will increase. Procedures may include those performed for gastrointestinal obstruction, acute appendicitis, or peritonsillar abscess. In these situations, an operation must be started regardless of rivaroxaban plasma concentration, to avoid further clinical complications. The decision for intraoperative hemostatic therapy is determined based on the extent of diffuse bleeding, which we describe later in this paper.

For an urgent but less critical indication for surgery, the timing of surgery should be considered in the same way as before an elective procedure:

- When was the last administration of rivaroxaban?
- Is the patient's renal function impaired?
- Is there an increased risk of perioperative bleeding associated with the surgical procedure?

Given the short half-life of rivaroxaban, after reaching the peak plasma level (~2–4 hours after dose) [8] the risk of drug-related bleeding will decrease with every hour interval between the last intake of rivaroxaban and surgery. In situations that constitute an urgent need for surgery, but that are also associated with an increased risk of bleeding (e.g. a large wound area), measurement of anti-Factor Xa levels (as described previously) might help to determine the timing of surgery, even if evidence-based data are lacking. If postponement of the operation is not possible, surgery may be required while the anticoagulant effect of rivaroxaban is still present. Owing to the risk of thromboembolism and the lack of data regarding efficacy in this setting, prophylactic administration of PCC is currently discouraged by some experts [31].

For vital operations in patients receiving rivaroxaban therapy, a sufficient supply of blood products and PCC, as well as an experienced surgical team (including an anesthesiologist), should be adequate in most cases as a first measure for bleeding control.

## 5. General Management of Bleeding Events

An algorithm for the management of bleeding in patients receiving the direct oral anticoagulants must take into account the severity of the bleeding, the primary cause, and localization, as well as the possibility of surgical hemostasis [32, 33].

According to the manufacturer, no clinically serious bleeding complications have been described with the use of rivaroxaban up to doses of 600 mg; this is because of a “ceiling effect” from limited absorption, with no further increase in average plasma exposure expected at supratherapeutic doses of 50 mg rivaroxaban or above [2].

In a patient with minor bleeding, delaying administration of the next dose or discontinuation of anticoagulation is recommended; the risk of thromboembolism should be considered when determining the duration of the discontinuation period [33]. The administration of prohemostatic agents or extensive laboratory analysis should not be required in this situation.

In phase III clinical trials, the risk of major or clinically relevant nonmajor bleeding with rivaroxaban was generally similar to that associated with VKAs or low molecular weight heparins. For the treatment of pulmonary embolism in the EINSTEIN PE study, rates of major bleeding with rivaroxaban were significantly lower than standard of care [11]. In general, some bleeding events may not be caused by anticoagulant use but rather are caused by preexisting or traumatically induced bleeding, which is increased in severity by anticoagulation. Consequently, therapeutic measures should be aimed at the cause and source of moderate to severe bleeding. If possible, mechanical compression or limited surgical or interventional care (e.g., vascular obliteration, suturing, gastroscopy, colonoscopy, coiling, and chemoembolization) is desirable. Further supportive measures such as blood transfusions and, depending on the severity of the bleeding, the administration of fresh frozen plasma, platelets, or intravenous antifibrinolytic agents [31, 34] (e.g., tranexamic acid as a bolus of 1000–2000 mg) may be used. This is because, with heavy blood loss, hemostatic and hemodynamic stabilization should be established to avoid the consequences of hemorrhagic shock.

**Figure 2:**
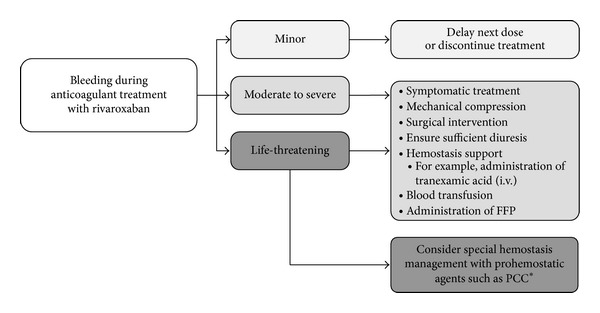
General algorithm for management of bleeding in patients receiving rivaroxaban [2, 31, 33]. *Clinical experience is limited with these agents. FFP = fresh frozen plasma; i.v. = intravenous; PCC = prothrombin complex concentrate.

Whether and how anticoagulation should be continued, based on the risk of recurrent bleeding and thromboembolism, can only be determined after bleeding has been successfully controlled. A general algorithm for the management of bleeding in patients receiving rivaroxaban is shown in Figure 2 [2, 31, 33].

## 6. Management of Life-Threatening Bleeding Events

Life-threatening bleeding events usually require special hemostasis management [35]. Because no laboratory test can exactly predict the likelihood of bleeding or hemostasis, a predominantly clinically oriented approach is indicated.

As a first step, life-threatening bleeding should be clinically defined and verified. Life- threatening bleeding is defined as a persistent need for transfusion or hemodynamic instability (e.g., a decrease in systolic blood pressure of 20% from baseline after administration of catecholamines) with the following bleeding localization: intracerebral hemorrhage; major bleeding in preformed body cavities (e.g., pleural, abdominal, or peritoneal); severe organ hemorrhage with impending organ failure; heavy compartmental bleeding, especially in the extremities; or major bleeding in the soft tissue of the neck with imminent respiratory compromise. The persistence of such bleeding would be assumed to lead to irreversible, severe damage to the patient; therefore, alleviation of the effects of rivaroxaban cannot be expected in these cases. Owing to its high plasma protein binding, hemodialysis of rivaroxaban is not possible [2].

In parallel, the potential impact of anticoagulation should be determined (when was rivaroxaban last administered? What is the patient's level of renal and hepatic function? Is there an intraoperative bleeding risk?), and the possible contribution of other causes of bleeding (including excessive fibrinolysis, defects in primary hemostasis, dilutional coagulopathy, hypothermia, acidosis) should be excluded or included. Therefore, it may be necessary to obtain at least one assessment of the effect of residual rivaroxaban. As described previously, a quantitative determination when used with appropriate rivaroxaban calibrators and controls [24]. If an anti-Factor Xa assay is not available, the emergency determination of PT with a rivaroxaban-sensitive reagent (e.g., STA Neoplastine CI Plus) may be suitable, although bleeding itself can affect the value of PT as well as other factors. If there is a significant deviation from the normal PT, an effect of rivaroxaban is likely to be present [31]. At this stage, prohemostatic agents, including PCCs, are a viable option. PCC preparations can be used immediately and have been shown to normalize thrombin generation after administration of rivaroxaban [36], which is mostly sufficient for clinical hemostasis.

The clinical evidence supporting effective reversal of direct Factor Xa inhibitors is currently limited [35]. PCCs comprise activated PCC (aPCC; FEIBA) or inactivated products containing three (Factors II, IX and X) or four (Factors II, IX, X and VII) clotting factors. The use of these agents may differ depending on country approvals. For example, only three-factor PCCs were licensed for use in the US until May 2013, when approval for four-factor PCCs was granted [37]. In European countries, as well as Australia and Japan, four-factor PCCs have been commercially available for a number of years. Studies in animal models [38, 39], as well as in healthy volunteers [36, 39, 40], demonstrate that PCCs reverse the anticoagulant effect of rivaroxaban effectively. PCCs have been suggested as a useful approach for rivaroxaban reversal in the case of severe, life-threatening bleeding [31, 34, 41]; however, a consensus has not been reached on their clinical benefits [42]. This stems from the lack of prospective data regarding clinical efficacy of PCC in patients. Dosages have not yet been clinically tested and some recommendations are based on a cross-section of the Board of the German Medical Association guidelines [35]. Recommendations for use of recombinant activated Factor VII (rFVIIa; NovoSeven), PCC, or aPCC might be found only in local prescribing information, or based on clinical experience with traditional anticoagulants. In addition, PCC preparations, which we consider to be potentially valuable for Factor Xa inhibition, are not approved in all countries.

**Figure 3:**
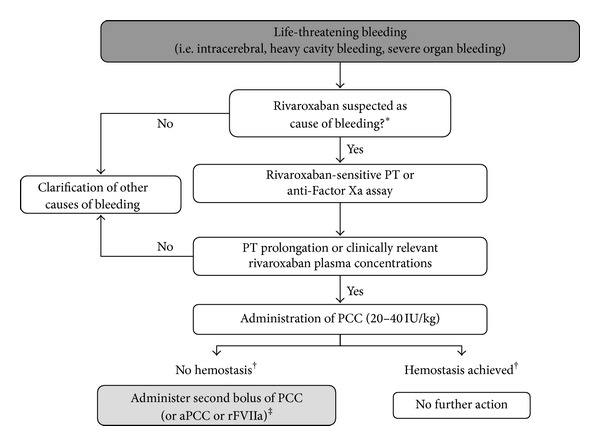
Special management of life-threatening bleeding in patients receiving rivaroxaban. *Refers to information including timing of last dose of rivaroxaban, dose administered, and risk factors for bleeding (i.e., renal/hepatic function and concomitant medication). ^†^After 10–15 minutes. ^‡^Prothrombotic risk may be higher with aPCC or rFVIIa. aPCC = activated prothrombin complex concentrate; PCC = prothrombin complex concentrate; PT = prothrombin time; rFVIIa = recombinant activated Factor VII.

Proposed guidance for the management of life-threatening bleeding with rivaroxaban is shown in Figure 3. A risk assessment should always be carried out during reversal of bleeding with the direct oral anticoagulants, particularly with regard to the potential thromboembolic risk. For the currently available PCC preparations, if the maximum amount is followed precisely (e.g., a 20–40 IU/kg bolus), a level of thrombotic risk might be expected; however, this would not be considered clinically relevant. The safety associated with PCC use has greatly improved compared with older PCC preparations, and pharmacovigilance data have shown no proven cases of thromboembolism [43]. For the use of rFVIIa in the so-called “off-label” indications, results of a meta-analysis showed an increased arterial thromboembolic rate with rFVIIa compared with placebo (4.5% versus 2.0%, resp.) [44]. When applying aPCC, a higher thromboembolic risk is likely, especially with multiple applications [45]. Therefore, neither rFVIIa nor aPCC is recommended for the management of severe bleeding in patients who have high thromboembolic risk. A combined use of these drugs should be avoided [46]. Any potential differences in thrombogenic risk between PCCs, rFVIIa, or aPCC have not yet been confirmed by clinical trials.

## 7. Conclusions

In patients receiving rivaroxaban who have normal renal and hepatic function, discontinuation of rivaroxaban at least 24 hours before surgery for an elective intervention is sufficient to minimize the risk of bleeding.

In the assessment of bleeding risk, clinical factors such as reduced renal or hepatic function and older age should be taken into consideration. Increased levels of rivaroxaban may be expected in these patients, who might have an increased risk of bleeding; therefore, increasing the drug-free period before surgery may need to be considered. In cases of mild bleeding, from a clinical perspective, intensive medical care of these patients is required and delay or temporary discontinuation of rivaroxaban is recommended. Coagulation testing may be helpful in several settings, including patients with known high-grade renal and hepatic failure, which cause a prolonged elimination time of rivaroxaban. Knowledge of the timing of the last rivaroxaban dose is important when scheduling such tests, which should also be performed and interpreted promptly owing to the short half-life of rivaroxaban. In life-threatening bleeding situations, in addition to maintaining hemodynamic stability, special hemostasis management is usually required [31, 47, 48].

After the assessment of factors associated with an increased risk of bleeding (which may include confirmation of other potential causes of bleeding, such as bleeding from wound surfaces, hyperfibrinolysis, primary hemostatic disorders, dilutional coagulopathy, hypothermia, and acidosis), the timing of the last dose as well as the administered dose of rivaroxaban is important. For measurement of rivaroxaban concentrations, an anti-Factor Xa chromogenic assay is regarded as a suitable. If a significant deviation in the measured PT (seconds) from the normal range is seen, a clinically relevant effect of rivaroxaban is likely [31].

Depending on the clinical situation and extent of the anticoagulant effect, the use of prohemostatic agents may be required. In the absence of clinical data, PCC appears to be a rational option for reversal of bleeding in patients receiving direct oral anticoagulants. In certain cases, rFVIIa or aPCC could be considered for prompt restoration of hemostasis, which should always be assessed clinically and not by a clotting test. The doses of these drugs have not been tested clinically and the risk of thromboembolism should be considered. Further studies of prohemostatic agents, as well as reversal agents of direct oral anticoagulants and heparins [49, 50] (or more specifically Factor Xa [49, 50]), are underway: these will provide valuable information on the efficacy and safety of this approach (if needed) in patients receiving therapy with a direct oral anticoagulant such as rivaroxaban.

## Figures and Tables

**Table 1:** Change of prothrombin time in seconds (5–95% percentile) using various rivaroxaban doses and the STA Neoplastine CI Plus Assay [2].

Indication	Dose	PT at maximum plasma concentration (hours after ingestion)	PT at minimum plasma concentration (hours after ingestion)
Prevention of VTE	10 mg od	13–25 s (2–4 h)	Not studied
Treatment of VTE (first 3 weeks)	15 mg bid	17–32 s (2–4 h)	14–24 s (8–16 h)
Treatment of VTE (after 3 weeks)	20 mg od	15–30 s (2–4 h)	13–20 s (18–30 h)
Nonvalvular AF for stroke prevention	20 mg od	14–40 s (1–4 h)	12–26 s (16–36 h)
Nonvalvular AF for stroke prevention (CrCl <50 mL/min)	15 mg od	10–50 s (1–4 h)	12–26 s (16–36 h)

AF: atrial fibrillation; bid: twice daily; CrCl: creatinine clearance; od: once daily; PT: prothrombin time; VTE: venous thromboembolism.
